# A review of the *Pseudobarbus afer* (Peters, 1864) species complex (Teleostei, Cyprinidae) in the eastern Cape Fold Ecoregion of South Africa

**DOI:** 10.3897/zookeys.657.11076

**Published:** 2017-02-20

**Authors:** Albert Chakona, Paul H. Skelton

**Affiliations:** 1 South African Institute for Aquatic Biodiversity, Private Bag 1015, Grahamstown 6140, South Africa

**Keywords:** Cape Fold Ecoregion, endemic hotspot, single barbeled redfins, *Pseudobarbus senticeps*, *Pseudobarbus swartzi*

## Abstract

The Eastern Cape redfin, *Pseudobarbus afer*, has long been considered to be a single widespread and variable species occurring in multiple isolated river systems in the Cape Fold Ecoregion (CFE) at the southern tip of Africa. Mitochondrial cytochrome *b* and control region sequence data of individuals from populations currently assigned to *Pseudobarbus afer* across the species’ distribution range revealed existence of four deeply divergent taxonomic units: (i) the Mandela lineage confined to the Sundays, Swartkops and Baakens river systems, (ii) the Krom lineage endemic to the Krom River system, (iii) the St Francis lineage occurring in the Gamtoos and adjacent river systems, and (iv) the Forest lineage occurring in several coastal river systems from the Tsitsikamma to the Klein Brak River system. The Forest lineage is closely related to *Pseudobarbus phlegethon* from the Olifants River system on the west coast of South Africa, suggesting that it does not belong to *Pseudobarbus afer* s.l. Herein we focus on the three lineages within the *Pseudobarbus afer* s.l. complex and provide new diagnosis for *Pseudobarbus afer* s.s (Mandela lineage), revalidate *Pseudobarbus senticeps* (Krom lineage) as a distinct species, and describe a new species *Pseudobarbus swartzi* (St Francis lineage). The three species exhibit subtle differences, which explains why they were previously considered to represent a single variable and widespread species. *Pseudobarbus senticeps* differs from both *Pseudobarbus afer* and *Pseudobarbus swartzi* by having fewer (i.e. larger) scales (25–33, mode 29 lateral line scale series; 10–12, mode 11 circumpeduncular scales) and presence of a lateral stripe which terminates in a conspicuous triangular blotch at the base of the caudal fin. Long barbels which reach or surpass the vertical through the posterior edge of the eye further separate *Pseudobarbus senticeps* from *Pseudobarbus afer* s.s. which possesses simple short barbels which do not reach the vertical through the posterior margin of the eye. *Pseudobarbus afer* s.s differs from *Pseudobarbus swartzi* **sp. n.** by possession of fewer scale rows along the lateral line (29–35, mode 32 *vs* 34–37, mode 36 in *Pseudobarbus swartzi*), fewer scales around the caudal peduncle (12–16, mode 12 *vs* 13–17, mode 16 in *Pseudobarbus swartzi*) and a distinct mesh or net-like pigmentation pattern on latero-ventral scales.

## Introduction

The cyprinid genus *Pseudobarbus* currently contains nine valid species endemic to southern Africa. All species of this genus are confined to streams associated with the Cape Fold Ecoregion (CFE) at the southern tip of Africa, with the exception of *Pseudobarbus quathlambae* which is endemic to the headwater tributaries of the Orange River in the Lesotho Highlands (Barnard 1943; Skelton 1988; Chakona and Swartz 2013; Chakona et al. 2014). Redfins were previously assigned to the genus *Barbus* Cuvier & Cloquet, 1816 until Skelton (1988) revalidated Smith’s (1841) subgenus Pseudobarbus and raised it to full generic status. The monophyly of *Pseudobarbus* is supported by both molecular data (mitochondrial DNA sequences) and morphological characters (Swartz et al. 2009). Species of this tetraploid genus (Naran et al. 2006) are characterised by presence of bright redfins, a soft or flexible primary dorsal spine and development of prominent nuptial tubercles in mature breeding males (Skelton 1988). Most recently Yang et al. (2015) suggested the genus *Pseudobarbus* be expanded to include all tetraploid cyprinines from southern Africa. Whilst this suggestion is accepted pending a critical evaluation of the generic status of these additional species (Skelton 2016), in this paper the traditional, more restricted lineage is intended.

**Figure 1.**
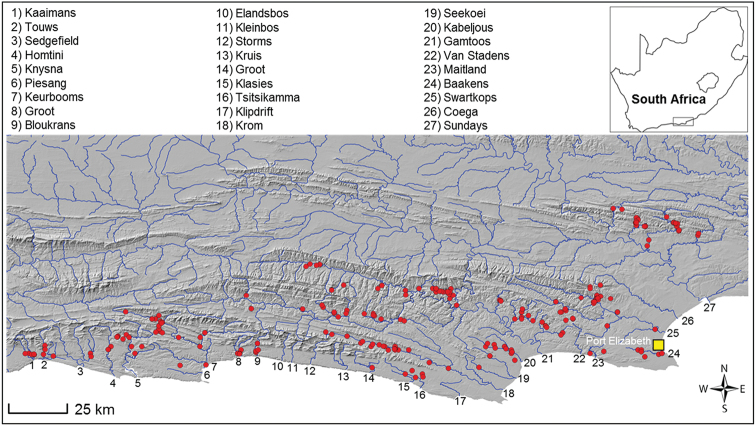
Distribution of the Eastern Cape redfin, *Pseudobarbus afer*, as presently described.

Many species of *Pseudobarbus* have restricted distribution ranges (Skelton 1988; Chakona and Swartz 2013; Chakona et al. 2014). *Pseudobarbus afer* (Peters, 1864) as it is currently described was considered to be the only exception as it has the widest distribution range of all redfin species. Its distribution spans across 28 isolated river systems from the Klein Brak which discharges into Mossel Bay to the Sundays which flows into Nelson Mandela Bay (also known as Algoa Bay) near Port Elizabeth (Figure 1). As with many other southern African freshwater fishes with broad geographical ranges, *Pseudobarbus afer* has had a long and confused taxonomic history. The three syntype specimens (ZMB 5413) were collected by Ludwig Krebs who had settled in the Port Elizabeth-Uitenhage area in the 1820’s (Ffolliot and Liversidge 1971). There are uncertainties regarding the type locality of *Pseudobarbus afer*, however, based on available evidence and discussions between Dr R. Liversidge and Dr R. A. Jubb, the Swartkops River system was suggested as the likely original locality of *Pseudobarbus afer* (see Jubb 1965). Smith (1936) described *Pseudobarbus senticeps* (as *Barbus senticeps*) based on the holotype collected from the Krom River system, near the central portion of the present distribution of *Pseudobarbus afer*. He identified the much longer barbels and the lower number of lateral line scales as the unique features that differentiated *Pseudobarbus senticeps* from *Pseudobarbus afer*. For a long period after being described, *Barbus afer* was not recognised as a ‘redfin’ species as its live colours were unknown. Jubb (1963, 1965) was the first to recognise that it was a ‘redfin’ minnow endemic to the Eastern Cape region. Jubb (1965) subsequently placed *Barbus senticeps* in synonymy with Peters’ *Barbus afer*. Boulenger (1911) described *Pseudobarbus asper* (as *Barbus asper*) from the Gamtoos river system based on four specimens collected from the Groot River near Steytlerville. The distinction between *Pseudobarbus afer* and *Pseudobarbus asper* was often uncertain, resulting in both Barnard (1943) and Jubb (1965) concluding that populations of *Pseudobarbus* from the southern coastal systems (Klein Brak, Kabeljous, Rondebosch and Keurbooms) belonged to *Pseudobarbus asper*. This decision was later revoked by Skelton (1988) who determined the southern coastal populations to be distinct from *Pseudobarbus asper* and he assigned them to *Pseudobarbus afer*. Skelton (1988) concluded that *Pseudobarbus asper* is restricted to the Gouritz and Gamtoos river systems.

The taxonomic integrity of the Eastern Cape redfin, *Pseudobarbus afer*, however remained unclear as Skelton (1988) reported considerable meristic variability across the species’ distribution range, mainly regarding differences in scale counts (a proxy for scale size). Skelton (1988) identified four subgroupings within *Pseudobarbus afer* based on lateral line scale counts: the western population distributed in the Knysna, Goukamma, Keurbooms, Groot and Bloukranz (mode 35), the Krom population (mode 29), the Gamtoos population (mode 36), and the fourth group comprising the Baakens, Swartkops and Sundays populations (mode 32) (Figure 1). He commented that the variation in scale counts for *Pseudobarbus afer* (25 to 45 lateral line series) was not consistent with that of any redfin species or most African cyprinids, suggesting that the Eastern Cape redfin could potentially be a complex of morphologically very similar species.

A molecular study by Swartz et al. (2007) identified four distinct lineages within *Pseudobarbus afer* (which he named as the Forest, Krom, St Francis and Mandela lineages) that are separated by deep mitochondrial cytochrome *b* (cyt *b*) genetic divergences (3.3–8.3%). The Forest lineage occurs in multiple coastal river systems from the Tsitsikamma River system to the Klein Brak River system. The Krom lineage is restricted to the Krom River system, which is the type locality of *Pseudobarbus senticeps* (Smith, 1936). The St Francis lineage occurs in the Gamtoos, Kabeljous and Swart River systems draining into St Francis Bay. The Mandela lineage is restricted to the Baakens, Swartkops and Sundays river systems draining into Algoa Bay, close to the city of Port Elizabeth (Swartz et al. 2007, 2009). The geographical distributions of these lineages were largely consistent with the subgroupings identified by Skelton (1988). The deep genetic differentiation among these lineages suggest that *Pseudobarbus afer* as currently recognised is a complex of two previously described species and at least two candidate taxa.

Herein we demonstrate that, in addition to their deep genetic divergence, the Krom, St Francis and Mandela lineages can be separated based on scale counts, length of oral barbels and consistent differences in body colour pattern, supporting their status as distinct species. As the specimens that were used for Peters’ (1864) description were likely collected from the Swartkops River system (Jubb 1965; Skelton 1988), we consider the Mandela lineage to represent *Pseudobarbus afer* s.s. Here we re-describe *Pseudobarbus afer* s.s. and review its geographic distribution, resurrect *Pseudobarbus senticeps* as valid for the Krom lineage, and delineate morphological differences supporting recognition of the St Francis lineage as a new species, which is described herein as *Pseudobarbus swartzi* sp. n. The Forest lineage was not included in the present study as a phylogenetic analysis based on combined morphological and molecular data indicated that it was more closely related to *Pseudobarbus phlegethon* from the Olifants River system on the west coast of South Africa (Swartz et al. 2009), suggesting that phylogenetically it does not belong to the *Pseudobarbus afer* s.l. complex. The taxonomic status of the Forest lineage will be addressed in a further study that will incorporate the more closely related *Pseudobarbus phlegethon*.

## Materials and methods

### Sample collection

Morphometric and meristic data used for the present study were generated from both historical collections obtained from the South African Institute for Aquatic Biodiversity (SAIAB) and fresh specimens that were collected between 2010 and 2016. The samples were collected using a combination of seine nets, hand nets and electrofishing. Surveys for the freshly collected specimens followed recommended ethics guidelines of SAIAB. At each locality, a subsample of the captured fishes was anaesthetised with clove oil and a small piece of muscle tissue was dissected from the specimens and preserved in 95% ethanol. Source specimens were fixed in 10% formalin. Upon returning to the laboratory, DNA tissues were stored at -20^o^ C and voucher specimens were transferred through 10% and 50% to 70% ethanol for long-term storage. The specimens were deposited in the National Fish Collection at SAIAB in Grahamstown, South Africa. The type material has been deposited at SAIAB and Royal Museum of Central Africa (MRAC).

### Molecular data

Fresh topotypic specimens for *Pseudobarbus afer* s.s. were collected from the Blindekloof River, a tributary of the Swartkops River system between 2010 and 2015. Topotypic specimens for *Pseudobarbus senticeps* were collected from the Assegaaibos River, a tributary of the Krom River system in 2014. Additional DNA tissue samples were taken from specimens collected from 10 localities in 2015 and 2016 to fill in sampling gaps in the Kouga and Groot sub-catchments of the Gamtoos River system. Methods for obtaining DNA sequence data and their analyses follow Swartz et al. (2007) and Chakona et al. (2013a). We sequenced the mitochondrial cytochrome *b* (cyt *b*) gene to compare with data generated from previous studies (Swartz et al. 2007; Chakona and Swartz 2013; Chakona et al. 2014). The sequences were assigned as topogenetypes (for *Pseudobarbus afer* and *Pseudobarbus senticeps*) and hologenetype and paragenetypes (for *Pseudobarbus swartzi* sp. n.) following Chakrabarty (2010). We included all the newly generated cyt *b* sequences (*n* = 30) to the genetic analysis done by Swartz et al. (2007) to show their phylogenetic position in relation to all known species and lineages of the single barbeled redfins in the CFE. All the new sequences were submitted to GenBank (accession numbers: KY472256–KY472285).

### Morphometric and meristic measurements

Methods used to obtain meristic and morphometric data (Table 1) follow Armbruster (2012), Chakona and Swartz (2013), Chakona et al. (2014) and Skelton (1988). Measurements were taken to the nearest 0.1 mm using electronic digital or dial callipers.

**Table 1.** Morphological characters of *Pseudobarbus* species used in the present study (reproduced from Chakona et al., 2014).

Character	Description	Acronym
*Morphometric measurements*		
Standard length	Tip of the snout to the point of flexure of the caudal fin	SL
Pre-dorsal length	Tip of the snout to the origin of the dorsal fin	PDL
Head length	Tip of the snout to the posterior bony margin of the operculum	HL
Snout length	Tip of the snout to the anterior bony edge of the orbit	S
Orbit diameter	The greatest bony diameter of the orbit	OD
Inter-orbit length	Straight line distance between the bony edges of the orbits	IO
Post-orbit length	Distance between the posterior bony edge of orbit to the posterior bony edge of operculum	PO
Head depth	Maximum depth measured from the nape	HD
Body depth	Maximum depth measured from the anterior base of the dorsal fin	BD
Anterior barbel length	From base to tip of anterior barbel	AB
Posterior barbel length	From base to tip of posterior barbel	PB
Dorsal fin base	Distance between anterior and posterior base of dorsal fin	DB
Dorsal fin height	From anterior base to tip of dorsal fin	DH
Pectoral fin length	From anterior base to tip of pectoral fin	PtL
Pelvic fin length	From anterior base to tip of pelvic fin	PvL
Anal fin base	Distance between anterior and posterior base of anal fin	AfB
Anal fin height	From anterior base to tip of anal fin	AfH
Caudal peduncle length	Distance from posterior base of anal fin the point of flexure of the caudal fin	CPL
Caudal peduncle depth	The least depth of the caudal peduncle	CPD
Pectoral to pelvic fin length	Distance between the posterior margins of the fin bases	PP
Pelvic to anal fin length	Distance between the posterior base of the pelvic fin to the anterior base of the anal fin	PA
Body width	The greatest width just anterior to the origin of the dorsal fin	BW
*Meristic counts*		
Lateral line scales	Number of scale rows along the lateral line	LL
Lateral line to dorsal fin scales	Number of scale rows between lateral line scale row and anterior base of the dorsal fin	LD
Lateral line to pelvic fin scales	Number of scale rows between lateral line scale row and base of pelvic fin	LP
Lateral line to anal fin scales	Number of scale rows between lateral line scale row and anterior base of the anal fin	LA
Caudal peduncle scales	Number of scale rows around the caudal peduncle	CP
Predorsal scales	Number of scale rows from the edge of the nape to the anterior base of the dorsal fin	PDS
Unbranched dorsal fin rays	Number of unbranched primary dorsal rays	UdR
Branched dorsal fin rays	Number of branched dorsal rays	BdR
Total vertebrae	Total number of vertebrae in vertebral column (including four Weberian vertebrae and a single ural centrum)	TV
Pre-dorsal vertebrae	Total number of vertebrae in advance of the leading dorsal fin pterygiophore (including the four Weberian vertebrae)	PdV
Pre-caudal vertebrae	Total number of vertebrae in advance of the first caudal vertebrae (i.e. the vertebrae opposite the leading anal pterygiophore) plus the four Weberian vertebrae	PcV
Pre-anal vertebrae	Total number of vertebrae in advance of the leading anal pterygiophore (including the four Weberian vertebrae)	PaV
Caudal vertebrae	Total number of vertebrae before the last precaudal vertebrae (including a single ural centrum)	CV

We included additional raw data from Skelton (1988) to determine the degree of morphological divergence within the *Pseudobarbus afer* s.l. complex. Specimens were assigned to three groups based on geographical origin and genetic results: *Pseudobarbus afer* s.s. (specimens from the Sundays, Swartkops and Baakens River systems; *n* = 68), *Pseudobarbus swartzi* sp. n. (specimens from the Gamtoos River system, *n* = 64) and *Pseudobarbus senticeps* (specimens from the Krom River system; *n* = 31).

Principal Component Analysis (PCA) was performed on raw meristic variables and morphometric variables in percentages as well as log transformed morphometric data to explore the separation of the specimens and identify the variables that contribute the most to differences among groups. Fin and barbel erosion was observed in some specimens, particularly those that were collected from polluted waters. Such specimens were excluded from the PCA for morphometric data. Morphometric and meristic data were analysed separately using the statistical program PAST (Hammer et al. 2001).

## Results

### Molecular data

**Figure 2.**
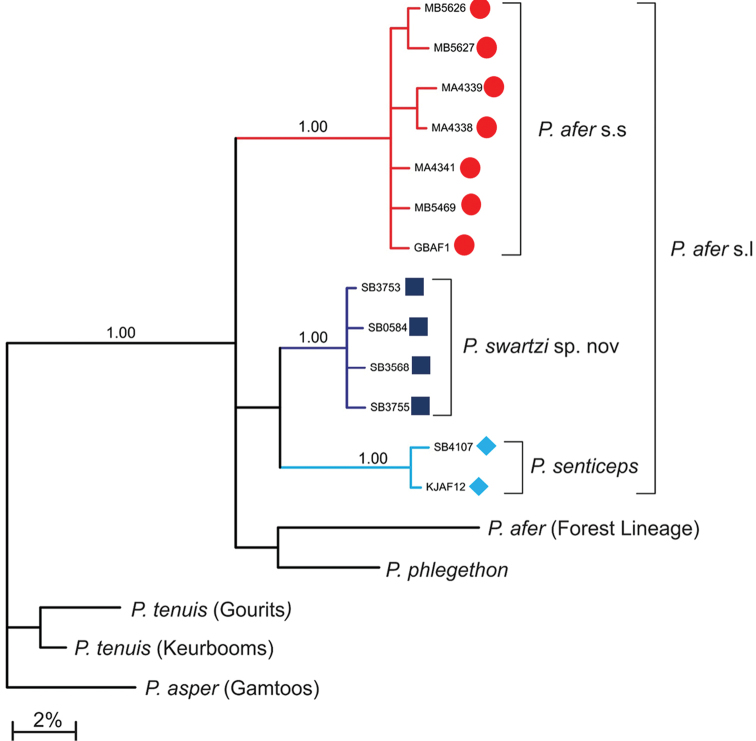
Bayesian phylogenetic tree showing genetic distances between *Pseudobarbus afer* s. s, *Pseudobarbus senticeps* and *Pseudobarbus swartzi* sp. n. and their relationships with the other single barbeled *Pseudobarbus* species and lineages in the Cape Fold Ecoregion of South Africa. Bayesian posterior probabilities are shown on the branches. The symbols correspond to the distribution map of the three species in Figure 7.

Consistent with results from previous studies, Bayesian phylogenetic analysis recovered four major clades within *Pseudobarbus afer* (Figure 2). Samples from the Swartkops and Sundays (*Pseudobarbus afer* s.s.) were closely related with 0.00–1.03% sequence divergence, but were 6.75–7.81% divergent from *Pseudobarbus senticeps* and 4.76–6.61% divergent from *Pseudobarbus swartzi* sp. n. Samples from the Krom River system (*Pseudobarbus senticeps*) were 0.00–0.20% divergent from each other, but were separated by 3.32–4.09% divergence from *Pseudobarbus swartzi* sp. n. Genetic divergence within *Pseudobarbus swartzi* sp. n. (samples from the Gamtoos, Kabeljous and Swart River systems) ranged from 0.00–0.40%. The ranges of genetic divergence values among *Pseudobarbus afer* s.s, *Pseudobarbus senticeps* and *Pseudobarbus swartzi* are comparable to typical interspecific divergences found between other single barbeled redfins in the CFE that possess a flexible primary dorsal spine (e.g. see Table 2 for divergence between *Pseudobarbus asper* and *Pseudobarbus tenuis*). *Pseudobarbus afer* s.s, *Pseudobarbus senticeps* and *Pseudobarbus swartzi* are also genetically differentiated from all currently described species or known genetic lineages of *Pseudobarbus* (Table 2), indicating that they are distinct taxonomic entities.

**Table 2.** Ranges of model-corrected genetic divergences (%) between species and lineages (in parenthesis) of the soft-rayed redfins of the genus *Pseudobarbus*.

	*afer* s.s	*swartzi*	*senticeps*	*asper*	*tenuis*	*phlegethon*	afer (forest)	*quathlambae*	*skeltoni*	burchelli (breede)	burchelli (heuningnes)	burchelli (tradou)	*burgi*	*verloreni*
*afer* s.s	0.00–1.03													
*swartzi*	4.76–6.61	0.00–0.40												
*senticeps*	6.75–7.81	3.32–4.09	0.00–0.20											
*asper*	8.59–10.12	8.25–8.89	9.87–10.23	–										
*tenuis*	5.94–8.97	5.86–7.37	6.69–9.23	3.04–4.25	0.00–1.88									
*phlegethon*	5.27–5.84	4.34–4.89	6.24–6.54	9.17	7.90–9.56	–								
*afer* (forest)	7.65–8.30	5.66–6.23	7.91–7.58	10.75	8.76–10.52	5.72	–							
*quathlambae*	19.43–22.06	19.28–21.54	20.20–22.48	21.23–22.91	18.71–20.48	20.80–21.59	19.75–21.59	0.00–2.34						
*skeltoni*	12.84–15.03	10.39–11.19	13.00–13.02	17.22–17.72	14.96–17.38	13.73–14.16	10.98–11.38	25.22 –27.79	0.00–0.20					
*burchelli* (breede)	6.80–8.48	6.00–6.89	8.08–8.72	9.77–10.10	7.86–9.82	8.10–8.40	8.36–8.68	16.79–17.47	9.92–10.65	0.00–0.19				
*burchelli* (heuningnes)	6.51–7.87	5.19–5.76	7.80–8.12	10.86	8.23–9.91	7.81	8.11	19.10–19.85	9.98–10.35	1.89–2.10	–			
*burchelli* (tradou)	6.90–8.29	6.70–7.03	8.30–8.64	10.47	8.07–9.65	9.61	8.28	16.05–16.66	9.49–9.87	4.10–4.35	4.39	–		
*burgi*	8.95–10.03	7.63–7.98	9.31–9.67	9.74	9.86–11.55	8.67	9.92	22.73–23.52	13.01–13.43	7.19–7.49	6.32	7.97	–	
*verloreni*	9.84–11.44	8.95–9.62	9.22–9.91	11.85–12.54	8.71–10.97	12.47–12.83	10.26–0.97	19.66–23.78	12.40–12.83	6.92–8.12	7.27–8.19	6.44–7.33	9.50–10.19	0.00–0.60

### Morphological data

**Figure 3.**
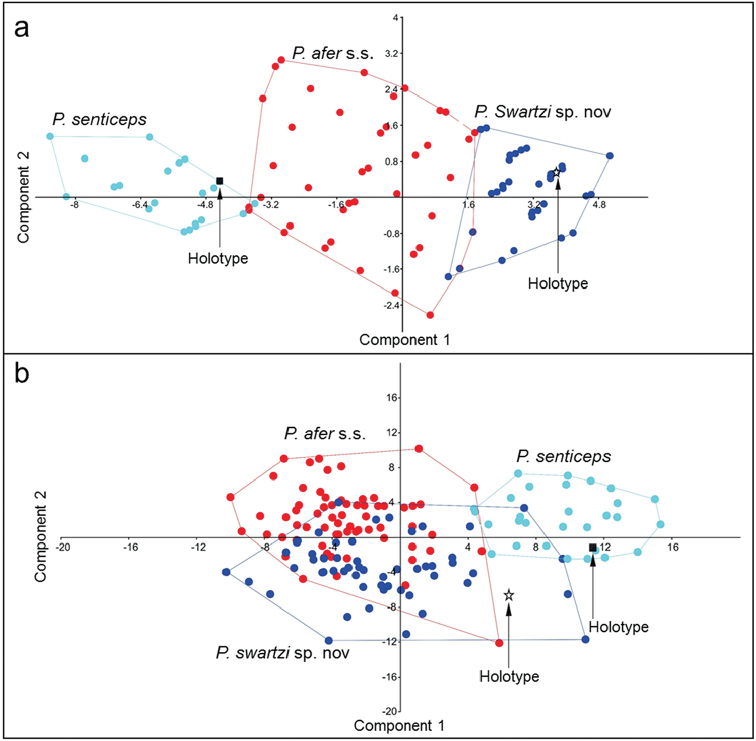
**a** Scatter plot of PC1 against PC2 for a PCA carried out on five raw meristic characters (scale counts) for 162 specimens of the *Pseudobarbus afer* complex **b** Scatter plot of PC1 against PC2 for a PCA carried out on 17 morphometric characters for 154 specimens of the *Pseudobarbus afer* complex. Syntypes were not included in the analyses as all three specimens are in very poor condition, with very few intact scales, flaccid bodies and damaged fins. The plots indicate that *Pseudobarbus senticeps*, *Pseudobarbus afer* s.s. and *Pseudobarbus swartzi* can be clearly separated based on scale counts, but the three species show considerable overlap in morphological characters.

**Figure 4.**
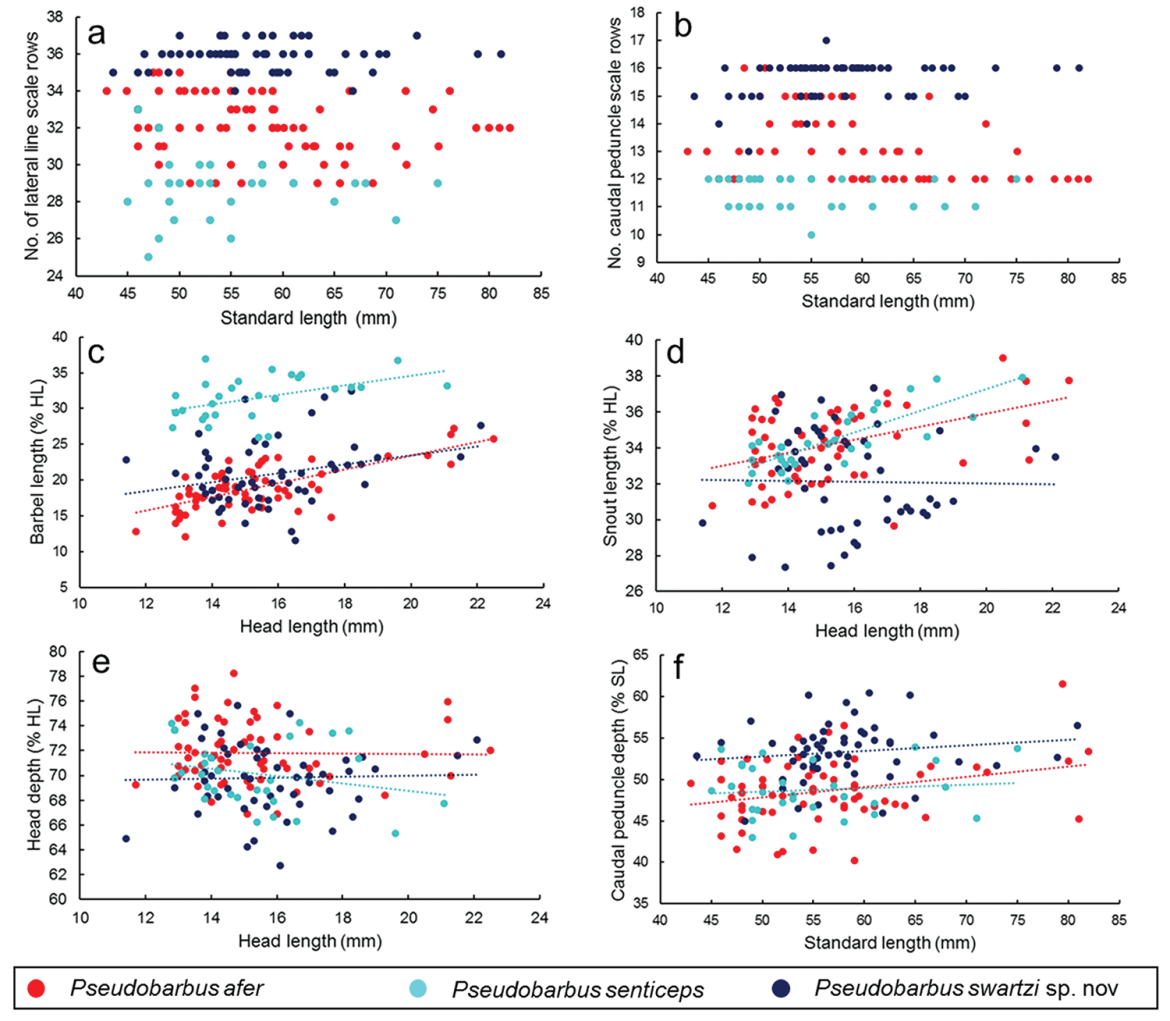
Scatter plots of scale counts and selected morphometric characters of *Pseudobarbus afer* s.s., *Pseudobarbus senticeps* and *Pseudobarbus swartzi* sp. n.

The first PCA performed on five meristics for 162 specimens of *Pseudobarbus afer* s.l. shows clear separation of *Pseudobarbus afer* s.s., *Pseudobarbus senticeps* and *Pseudobarbus swartzi* sp. n. based on scale counts (Figure 3a). The first Principal Component axis (PCI), primarily contrasting differences in the number of scale rows along the lateral line, explained 87.2% of the total variation (Table 3). The second PCA axis (PCII), primarily contrasting differences in the number of scale rows around the caudal peduncle, explained 9.0% of the total variation (Table 3). Specimens of *Pseudobarbus swartzi* sp. n. were associated positively with PCI (Figure 3a), describing individuals with a higher number of scales along the lateral line (mode = 36; range = 34–37; Table 4). Specimens of *Pseudobarbus senticeps* which are situated on the negative part of PCI have fewer scale rows along the lateral line (mode = 29; range = 25–33) compared to *Pseudobarbus afer* s.s and *Pseudobarbus swartzi* (Table 4). Note that the holotype of *Pseudobarbus senticeps* is not conspecific with *Pseudobarbus afer* s.s and *Pseudobarbus swartzi*. Scatterplots of scale counts against standard length show that *Pseudobarbus senticeps* and *Pseudobarbus swartzi* can be clearly separated based on differences in the number of scale rows along the lateral line and circumpeduncular scales (Figure 4a, b). Specimens of *Pseudobarbus afer* s.s. have intermediate meristic counts (lateral line scale rows and circumpeduncular scales) compared to *Pseudobarbus senticeps* and *Pseudobarbus swartzi*, with only a few individuals showing some overlap between *Pseudobarbus afer* and the other two species. Of the 31 specimens of *Pseudobarbus senticeps* examined for this study, only two had more than 30 scale rows along the lateral line, while only two of the 64 specimens of *Pseudobarbus swartzi* had 34 scale rows along the lateral line, and only five (out of 68) specimens of *Pseudobarbus afer* s.s had 35 scale rows along the lateral line.

**Table 3.** Factor loadings for the first two principal component (PC) axes of a PCA carried out on five meristic characters from 162 specimens of the *Pseudobarbus afer* complex. The most important factor loadings are in bold.

	PCI	PCII	PCIII
Eigenvalue	12.105	1.251	0.284
% Variance	87.20	9.01	2.05
Lateral line scale series	**0.857**	-0.494	0.131
Lateral line to dorsal fin scale rows	0.143	0.143	-**0.727**
Lateral line to pelvic fin scale rows	0.134	0.034	-0.491
Lateral line to anal fin scale rows	0.150	0.080	-0.386
Circumpeduncular scale rows	0.453	**0.853**	0.254

**Table 4.** Morphometric and meristic data for *Pseudobarbus afer* s.s, *Pseudobarbus senticeps* and *Pseudobarbus swartzi* sp. n.

	*Pseudobarbus afer*		*Pseudobarbus senticeps*		*Pseudobarbus swartzi* sp. n.	
	syntypes^‡^ *n* = 3	other specimens including topotypes *n* = 68	holotype	topotypes *n* = 29	holotype	other specimens including paratypes *n* = 63
Standard length (SL) (mm)	79.1–89.0	43.0–82.0	67.0	45.0–79.3	81.1	46.5–75.2
Head length (HL) (mm)	22.4–26.7	11.7–22.5	18.2	12.8–21.1	22.3	12.8–21.2
Percentage of SL (%)						
Head length	24.1–30.0	25.4–28.9	27.2	26.1–29.6	27.5	26.6–30.0
Predorsal length	51.2–55.0	49.6–55.5	50.7	49.3–55.0	53.8	53.3–56.6
Dorsal fin base	11.4–12.4	11.0–14.4	14.9	11.0–14.9	12.7	10.8–13.4
Dorsal fin height	–	20.4–25.2	25.4	20.8–25.4	24.9	23.5–27.8
Body depth	24.3–27.9	22.5–31.6	28.7	22.9–28.7	26.1	22.6–25.9
Body width	10.8–13.2	11.9–20.2	13.9	13.9–19.3	17.8	14.6–17.7
Caudal peduncle length	23.4–24.9	23.0–27.6	26.0	22.5–26.2	23.4	22.2–25.4
Percentage of HL (%)						
Head depth	61.8–70.7	66.5–78.2	73.6	65.3–74.3	72.6	63.7–71.6
Inter-obit	27.7–34.8	25.2–33.0	30.8	27.1–33.0	34.5	25.7–31.2
Snout length	24.0–29.3	29.7–39.0	34.6	29.8–37.9	33.6	28.9–34.0
Post orbit	40.8–49.7	42.2–51.2	52.7	44.2–52.7	50.7	44.6–47.7
Posterior barbel length	16.4–23.6	12.1–27.2	33.0	26.0–37.0	27.8	26.7–39.9
Orbit diameter	21.7–29.5	21.7–30.4	25.8	23.5–29.5	25.6	23.0–27.7
Percentage of caudal peduncle length (%)						
Caudal peduncle depth	43.2–50.9	40.3–61.5	52.3	43.0–54.5	57.9	43.6–54.3
Unbranched dorsal fin rays	ii–iii	iv (iii–iv)	iii	iv (iii–iv)	iii	iii
Branched dorsal fin rays	7	7 (6–7)	7	7	7	7
Unbranched anal fin rays	iii	iii	iii	iii	iii	iii
Branched anal fin rays	5	5	5	5	5	5
Pectoral fin rays	13–14	15 (14–17)	14	14 (13–15)	13	14 (12–14)
Pelvic fin rays	8	8 (8–9)	8	8 (8–9)	7	8 (7–8)
Lateral line scales	29–33	32 (29–35)	29	29 (25–30)	36	36 (35–37)
Lateral line to dorsal fin scale rows	4	5 (4–6)	5	5 (4–5)	6	6 (6–7)
Lateral line to pelvic fin scale rows	–	4 (3–5)	4	4 (3–4)	5	5 (4–5)
Lateral line to anal fin scale rows	–	4 (3–5)	4	3 (3–4)	5	5
Caudal peduncle scale rows	12–14	12 (12–16)	12	12 (10–12)	16	16 (15–16)
Predorsal scale rows	13–15	15 (14–16)	14	15 (12–15)	16	17–18 (16–20)
Total vertebrae		37 (36–39)		37 (35–38)	37	37 (37–38)*
Precaudal vertebrae		19 (18–20)		19 (18–19)	19	20 (19–20)*
Caudal vertebrae		18 (17–19)		18 (16–18)	18	18 (17–18)*
Predorsal vertebrae		12 (11–13)		12 (11–13)	13	13 (12–13)*

^‡^all three specimens are in very poor condition, with very few intact scales, flaccid bodies and damaged fins. *counts based on radiographs of the holotype and 12 paratypes

A PCA performed on 17 morphometric characters shows complete overlap between *Pseudobarbus afer* and *Pseudobarbus swartzi*, with marginal separation of *Pseudobarbus senticeps* from these two species (Figure 3b). The first four PCA axes, primarily contrasting differences in barbel length (PCI), caudal peduncle depth (PCII), snout length (PCIII) and head depth (PCIII & IV) explained 76.6% of the total variation (Table 5). Scatter plots of these four morphometric characters are presented in Figure 4c–f. *Pseudobarbus senticeps* is clearly differentiated from *Pseudobarbus afer* based on barbel length (Figure 4c). The three species show considerable overlap in snout length, head depth and caudal peduncle depth (Figure 4d–f), indicating that these characters are taxonomically uninformative to differentiate these species.

**Table 5.** Factor loadings for the first two principal component (PC) axes of a PCA carried out on morphometric characters from 154 specimens of the *Pseudobarbus afer* s.l. complex. The most important factor loadings are in bold.

	**PCI**	**PCII**	**PCIII**	**PCIV**
Eigenvalue	38.3	19.5	11.7	5.8
% Variance	38.9	19.9	11.9	5.9
Head length	0.053	-0.024	0.132	-0.030
Predorsal length	0.009	-0.156	0.261	0.154
Dorsal fin base	0.008	0.019	-0.160	-0.048
Dorsal fin height	0.031	-0.078	0.237	0.217
Anal fin base	0.020	-0.009	-0.106	-0.024
Body depth	-0.010	-0.145	-0.195	0.098
Body width	0.069	-0.075	-0.041	-0.001
Caudal peduncle length	-0.039	0.164	-0.034	0.087
Caudal peduncle depth	0.006	-**0.927**	-**0.21**1	-0.106
Posterior barbel	**0.986**	**0.02**2	-0.018	0.052
Pectoral to pelvic	-0.020	0.076	-0.290	-0.187
Pelvic to anal	0.000	0.007	-0.073	-0.088
Head depth	-0.050	0.049	-**0.590**	**0.712**
Snout length	0.039	0.201	-**0.517**	-**0.49**6
Orbit diameter	-0.046	0.085	-0.019	0.253
Post orbit	0.089	-0.003	-0.015	0.169
Inter orbit	0.052	-0.002	-0.175	-0.073

The first three axes of a PCA performed on log transformed data explained 89.4% of the total variation in the data set, but there was no separation of the species along PCI and PCII (results not shown). *Pseudobarbus senticeps* was completely separated from both *Pseudobarbus afer* and *Pseudobarbus swartzi* along PCIII (results not shown), with barbel length loading heavily on this axis (0.012 eigenvalue, 9.3% of total variation, 0.789 factor loading). Specimens of *Pseudobarbus senticeps* were positively associated with PCIII, describing individuals characterised by relatively long barbels, a pattern which is similar to the one revealed using percentage data described above. In this paper, we have thus only presented PCA scatter plots recovered from analysis of percentage data.

### Taxonomic accounts

#### Pseudobarbus afer

Taxon classificationAnimaliaTeleosteiCyprinidae

(Peters, 1864)

**Figure 5.**
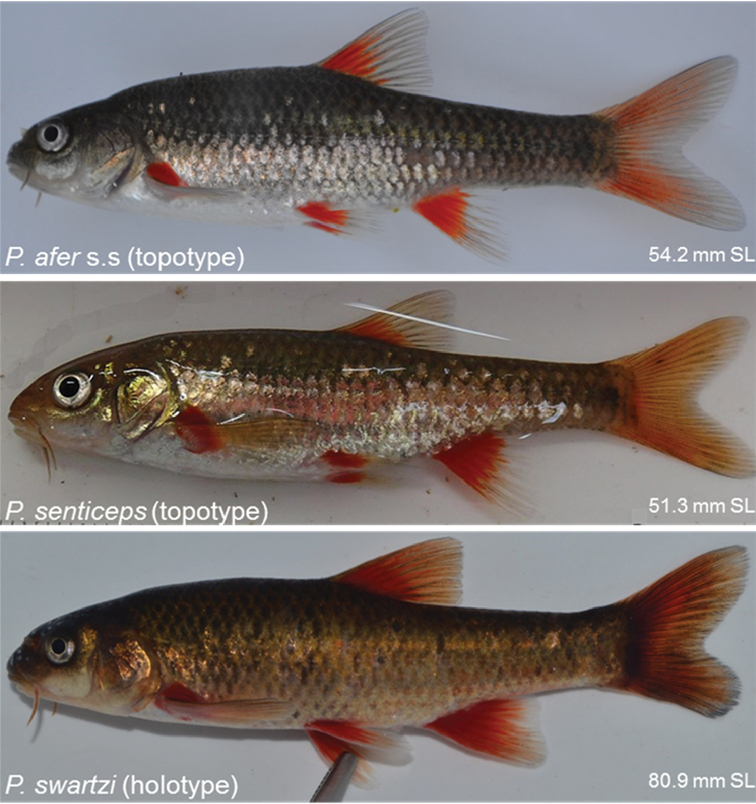
Live colours of *Pseudobarbus afer* s.s (SAIAB 203790) from the Waterkloof River, Swartkops River system, *Pseudobarbus senticeps* (RS17AL01) from the source pool in the Upper Krom River system and *Pseudobarbus swartzi* sp. n. (SAIAB 203792) from a tributary of the Wabooms River, Gamtoos River system.

**Figure 6.**
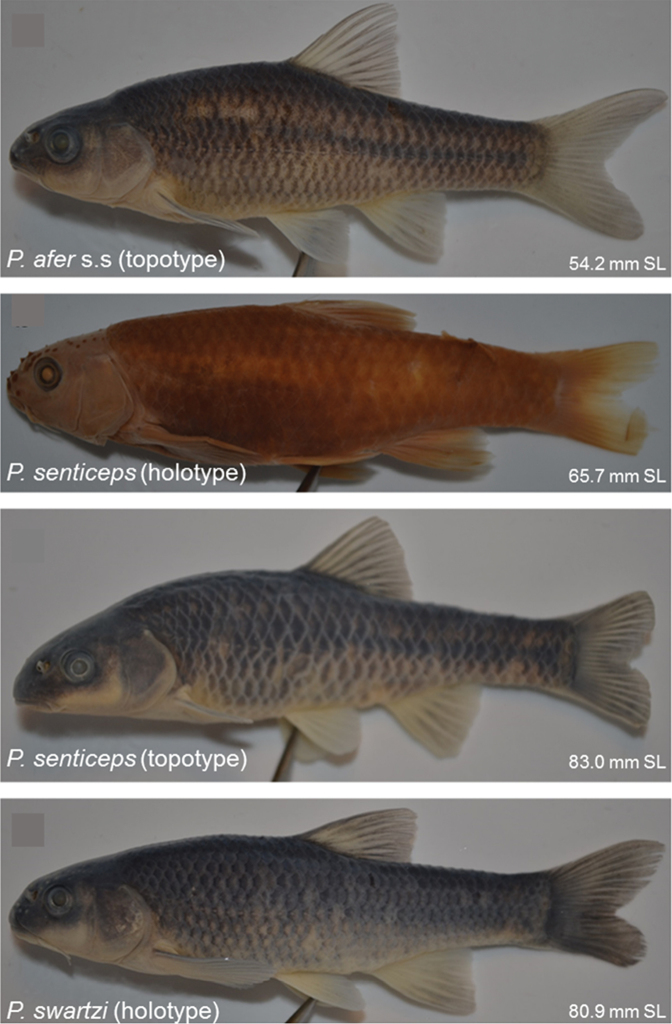
Preserved colours of *Pseudobarbus afer* s.s topotype (SAIAB 203790) from the Waterkloof River, Swartkops River system, *Pseudobarbus senticeps* holotype (SAIAB 304) from the Assegaaibosch River, Krom River system, *Pseudobarbus senticeps* topotype (SAIAB 200302) from the Assegaaibosch River, Krom River system, and *Pseudobarbus swartzi* sp. n. holotype (SAIAB 203792) from a tributary of the Wabooms River, Gamtoos River system. Note the differences in the arrangement of melanophores which produces distinct patterns on the latero-ventral scales of the three species.

**Figure 7.**
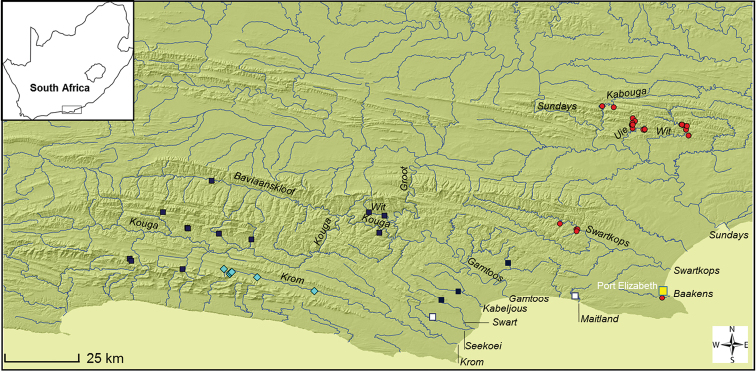
Map of the eastern Cape Fold Ecoregion showing confirmed distributions of *Pseudobarbus senticeps* (turquois diamonds) restricted to the Krom River system), *Pseudobarbus swartzi* sp. n. (blue squares) (restricted to the Gamtoos River system and the Kabeljous and Seekoei Rivers) and *Pseudobarbus afer* s.s (red circles)(Baakens, Swartkops and Sundays River systems) based on recent surveys (2000–2016). Additional surveys are required to more accurately map the distribution ranges of these species in the Krom, Gamtoos, Swart, Kabeljous, Baakens and Sundays, and determine the status of populations in the Seekoei and Maitland River systems (open squares).

Figures 5a , 6a , 7a

Barbus (Capoeta) afer Peters, 1864; Günther 1868; non Boulenger 1911; non Gilchrist and Thompson 1913; Barnard 1938, 1943; Jubb, 1965, 1967.
Barbus anoplus (non Weber, 1897): Boulenger 1911; Gilchrist and Thompson 1917.
Barbus vulneratus (non Castelnau, 1861): Gilchrist and Thompson 1913 (in part, species from the Baakens and Swartkops Rivers).
Barbus senticeps Smith, 1936; Barnard 1938, 1943.
Barbus asper non Boulenger, 1911: Barnard 1943; Jubb 1965; Smith and Smith 1966.
Pseudobarbus afer : Skelton 1988 in part (distributed from the Baakens to the Sundays River systems).

##### Syntypes.

ZMB 5413, 3 unsexed, 78, 89, 92 mm SL, original locality uncertain, but probably the Swartkops River system (Jubb 1965).

##### Topotypic specimens.

SAIAB 203790, *Pseudobarbus afer*, 8 unsexed, 42.1–54.2 mm SL, Waterkloof River, -33.7149528S, 25.2783833E, Groendal Wilderness, Swartkops River system, collected by A Chakona, W Kadye and B Ellender, 4 March 2015; SAIAB 97364, 2 males, 76.2–78.7 mm SL, Groendal Wilderness, Swartkops River system, -33.7000S, 25.2800E, collected by ER Swartz and B Ellender, 20 April 2010; SAIAB 97366, *Pseudobarbus afer*, 6 unsexed (31.9–70.8 mm SL), Groendal Wilderness, Swartkops River system, -33.7000S, 25.2900E, collected by B Ellender, 13 April 2010.

##### Etymology.

*afer* means African (citizen).

##### Diagnosis.

*Pseudobarbus afer* differs from *Pseudobarbus burchelli, Pseudobarbus burgi, Pseudobarbus skeltoni and Pseudobarbus verloreni* by possession of a single pair of oral barbels. Possession of fewer and larger scales separates *Pseudobarbus afer* (29–35, mode 32 scale rows along the lateral line) from *Pseudobarbus quathlambae* (> 60 scale rows along the lateral line) and *Pseudobarbus asper* (> 35 scales along the lateral line). Lack of a mid-dorsal stripe and a relatively deeper head and body profile separates *Pseudobarbus afer* (mean head depth: 71.8 % HL (range: 66.5–78.2%); mean body depth: 25.3% SL (range: 22.5–31.6%) from the more slender bodied *Pseudobarbus tenuis* (average head depth: 65.9 % HL (range: 61.1–71.2%); average body depth: 22.4 %SL (range: 18.8–26.8%)). Lack of prominent black spots and patches on the body distinguishes *Pseudobarbus afer* from *Pseudobarbus phlegethon*. *Pseudobarbus afer* most closely resembles *Pseudobarbus senticeps, Pseudobarbus swartzi* sp. n., and *Pseudobarbus asper*. Barbel length and the number of scale rows along the lateral line separates *Pseudobarbus afer* from these three species. Short barbels which do not reach the vertical through the posterior margin of the eye and a higher number of lateral line scales (29–35, mode 32) distinguishes *Pseudobarbus afer* from *Pseudobarbus senticeps* whose barbels reach or surpass the vertical through posterior edge of eye and has fewer and larger scales (lateral line scales 25–30, mode 29; caudal peduncle scales 10–12, mode 11; Figure 4a–c). *Pseudobarbus afer* further differs from *Pseudobarbus senticeps* by lack of a blotch of pigment at the base of the caudal fin, while the lateral stripe in *Pseudobarbus senticeps* terminates in a triangular blotch at the base of the caudal fin (Figure 6a). *Pseudobarbus afer* differs from *Pseudobarbus swartzi* sp. n. by possession of fewer scale rows along the lateral line (29–35, mode 32 *vs* 34–37, mode 36 in *Pseudobarbus swartzi*; Figure 4a) and fewer scales around the caudal peduncle (12–16, mode 12 *vs* 13–17, mode 16 in *Pseudobarbus swartzi*; Figure 4b). *Pseudobarbus afer* has a distinct mesh-like pigmentation pattern below the lateral line which further separates this species from *Pseudobarbus swartzi* which lacks discernible pigmentation pattern on the latero-ventral scales (Figure 6a, d). *Pseudobarbus afer* s.s is separated from *Pseudobarbus asper* by possession of fewer and larger scales (lateral line scale series 29–35, mode 32 *vs* 35–45, mode 37–40; caudal peduncle scale rows 12–16, mode 12 *vs* 16–22, mode 18–20).

##### Description.

Morphometric and meristic data summarised in Table 4 are based on three syntypes and 68 specimens (43–82 mm SL) from the Sundays, Swartkops and Baakens River systems. General body shape and colouration are shown in Figures 5a, 6a, 7a.

Body fusiform, more or less laterally compressed, with dorsal profile generally more convex than ventral profile. Body deepest around the anterior bases of the dorsal and pelvic fins. Caudal peduncle length is almost twice its depth. Head length sub-equal to body depth, snout slightly blunt, mouth sub-terminal and sickle shaped, with a single pair of simple short maxillary barbels. Barbel length shorter than orbit diameter, barbels do not surpass the vertical through posterior margin of pupil. Eyes moderately large, located dorsolaterally, closer to tip of snout than to the posterior margin of gill cover, orbit diameter shorter than snout length.

*Tuberculation*. Mature breeding males develop large conical tubercles on the snout and head dorsum. The bilateral clusters on the snout include 2–4 tubercles. Scattered tubercles on dorsal surface of head smaller than those on the snout. Minute tubercles develop in bands on the dorsal surface of pectoral fin rays and a row along the free edge of latero-dorsal scales.

*Scales*. 29–34 scale rows along the lateral line to end of hypural plate (point of flexure), 1–2 more scales to base of caudal fin. Of the 68 specimens examined, only 13 had 29–30 scale rows along the lateral line and only five specimens had 35 lateral line scale rows. The rest of the specimens (50) had 31–34 scale rows along the lateral line. Four to six scale rows between lateral line and anterior base of dorsal fin (dorsal fin origin), 3–5 scale rows between lateral line and pelvic fin origin, 3–5 scale rows between lateral line and anal fin origin, 14–16 pre-dorsal scale rows (from posterior edge of head to anterior base of dorsal fin), 12–16 circumpeduncular scales. Triangular naked patch between the gill covers and anterior base of pectoral fins, ventral scales between pectoral fin origin and pelvic fin origin reduced and embedded. Axillary scales of pelvic fin not prominent or elongate. Scales between posterior edge of head and dorsal fin origin embedded and smaller than flank scales.

*Fins*. Dorsal fin situated almost in the centre of the body (excluding caudal fin), origin slightly behind vertical through origin of pelvic fin, with 3–4 unbranched rays and 6–7 branched rays, distal margin straight to slightly concave, tip of depressed dorsal fin reaches within 1–2 scales to vertical through posterior base of anal fin. Pectoral fins fan-shaped, larger in males than females, with 14–17 rays, often reaches and surpasses base of pelvic fin in males, reaches 2 scales to base of pelvic fin in females. Pelvic fin with 8–9 rays, origin slightly in front of dorsal fin origin, tip of depressed pelvic fin does not reach anterior origin of anal fin, except in mature males. Anal fin with 3 simple rays and 5 (rarely 6) branched rays, distal margin almost straight to slightly convex, origin closer to anterior base of pelvic fin than caudal fin base. Caudal fin is obtusely forked, with a mode of 10+9 principal rays.

*Osteology*. Total vertebrae including Weberian apparatus 36–39 (mode 37), predorsal vertebrae including Weberian apparatus 11–13 (mode 12), precaudal vertebrae including Weberian apparatus 18–20 (mode 19), caudal vertebrae including Weberian apparatus 17–19 (mode 18).

*Colouration* (*live and fresh specimens)*. Refer to Figure 5a for general live colouration. Dorsum grey-black, sides and belly silvery white, base of fins bright red in adults. Vague dark mid-lateral band from behind the head to the base of the caudal fin.

*Colouration (preserved)*. The bright red pigmentation on base of fins and silvery colouration fades in preservative (Figure 6a). Dorsum and flanks above lateral line dark greyish. Belly off-white to yellow in most specimens. Band of pigment around centre of scales, basal segment of exposed area of scales without pigment, leaving a lighter band along distal edges of scales, producing a mesh or net-like pattern which is more pronounced on dorso-lateral scales (Figure 6a).

##### Distribution.

*Pseudobarbus afer* s.s. (referred to as the ‘Mandela lineage’ by Swartz et al. 2007) occurs in three isolated river systems (the Sundays, Swartkops and Baakens) which discharge into Algoa Bay near Port Elizabeth (Figure 7). Remnant populations of this species are highly fragmented, persisting in only a few less degraded upland tributaries that have not been invaded by alien species (Ellender et al. 2011).

##### Conservation status.

Once common and widely distributed throughout the Sundays, Swartkops and Baakens River systems, *Pseudobarbus afer* suffered severe decline in distribution and abundance, mainly due to invasion by alien predators and competitors, deterioration of water quality and loss of critical habitat. Consequently, this species (referred to as *Pseudobarbus afer* (Peters, 1864) by Tweddle et al. 2009) was listed as Endangered during the most recent IUCN assessment of the status and distribution of freshwater fishes in southern Africa (Tweddle et al. 2009). Invasion by alien fish was identified as the single most important threat to this species. Long-term persistence of this species in the Baakens River system is uncertain as the entire catchment of this system has been heavily urbanised and the river is now heavily infested with non-native species, particularly *Tilapia sparrmanii* and *Pseudocrenilabrus philander* (personal observations, March 2014). There is urgent need for comprehensive surveys to determine the status of the Baakens population and identify measures to prevent its eradication. The conservation status of *Pseudobarbus afer* s.s is being reassessed as part of a national program that is assessing the conservation status of all freshwater fishes of South Africa.

#### Pseudobarbus senticeps

Taxon classificationAnimaliaTeleosteiCyprinidae

(Smith 1936)

Figures 5b , 6b,c

Barbus senticeps Smith, 1936.
Barbus afer : Jubb 1963, 1965.
Pseudobarbus afer : Skelton 1988.

##### Holotype.

SAIAB 304, male, 65.7 mm SL, Assegaaibosch River, Krom River system.

##### Topotypic material.

SAIAB 200302, 9 unsexed, 23–83 mm SL, Assegaaibos River, Krom River system, -33.9452778S, 24.3139167E, collected by R Bills, V Bills and D Naran, 12 August 2014. SAIAB 121815 (formerly AMG 2651), 29 unsexed, 45–75 mm SL, Assegaaibosch River, -33.9413889S, 24.3188889E, Krom River system, collected by PH Skelton and J Stephenson, 20 January 1975.

##### Etymology.

‘*senticeps*’ refers to the thorny or prickly appearance of the head (*sentis* a thorn, bramble, and *ceps*, head) of sexually mature males due to the development of distinctly pointed tubercles on the snout, along the inner edges of the nares and orbits and head dorsum (see Figure 6b).

##### Diagnosis.

Possession of a single pair of oral barbels separates *Pseudobarbus senticeps* from *Pseudobarbus skeltoni*, *Pseudobarbus verloreni*, *Pseudobarbus burgi* and *Pseudobarbus burchelli*. Possession of fewer and larger scales separates *Pseudobarbus senticeps* (25–30, mode 29) from *Pseudobarbus quathlambae* (> 60 scale rows along the lateral line), *Pseudobarbus asper* (35–45; mode 37–40), *Pseudobarbus swartzi* sp. n. (34–37, mode 36) and *Pseudobarbus tenuis* (32–37, mode 35–36). There is overlap (although uncommon) in lateral line scale series between *Pseudobarbus senticeps*, *Pseudobarbus afer* (29–35, mode 32) and *Pseudobarbus phlegethon* (29–37, mode 35). A lateral stripe which terminates in a triangular blotch at the base of the caudal fin and longer barbels (reaching or surpassing vertical through the posterior edge of the eye) further separate *Pseudobarbus senticeps* from *Pseudobarbus afer* (barbels do not surpass vertical through the centre of the eye). Body colour pattern distinguishes *Pseudobarbus senticeps* from *Pseudobarbus phlegethon*. *Pseudobarbus phlegethon* is characterised by prominent black spots and patches on the body, which are lacking in *Pseudobarbus senticeps*.

##### Description.

Morphometric and meristic data summarised in Table 4 are based on the holotype and 29 topotype specimens (45–79.3 mm SL). General body shape and colouration are shown in Figures 5b, 6b, c.

A moderately laterally compressed, fusiform species. Cross-section of body between pectoral and pelvic fins ellipsoid. Dorsal profile of body, in lateral view, convex from snout tip to dorsal fin origin, straight and descending from dorsal fin origin to caudal fin insertion. Ventral profile, in lateral view, more or less straight or slightly convex from snout tip to anal fin origin, slightly concave and ascending from origin of anal fin to caudal fin insertion. Body deepest around anterior bases of dorsal and pelvic fin origins, progressively becoming narrower from anal fin origin towards the caudal fin. Caudal peduncle length almost twice as its depth, cross-section ellipsoid. Snout blunt or obtusely pointed. Mouth terminal, sickle shaped, its corner not reaching vertical through anterior margin of eye. A single pair of well developed, long and slender maxillary barbels present, barbel length longer than orbit diameter in most specimens. Eyes moderately large (23.5–29.5% HL), dorso-laterally positioned, and located closer to tip of snout than posterior margin of gill cover. Orbit diameter shorter than snout length.

*Tuberculation*. Mature breeding males develop prominent conical tubercles on the snout, along the nares and dorsal edges of the eyes. Bilateral clusters on snout include 2–4 tubercles in mature ripe males. Smaller, scattered tubercles develop on the head dorsum. A band of fine tubercles along dorsal surface of each of several anterior pectoral fin rays of mature breeding males.

*Scales*. Lateral line with 25–30 scales to end of hypural plate, 1–2 more scales to base of caudal fin. Four to five scale rows between lateral line and dorsal fin origin, 3–4 rows between lateral and pelvic fin origin, 3–4 scale rows between lateral line and anal fin origin, 12–15 pre-dorsal scale rows, 10–12 circumpeduncular scales. Breast scales reduced and embedded, giving a naked appearance to the region between the isthmus and base of pelvic fins. Elongated or triangular pelvic axillary scales absent. Scales between the nape and dorsal fin origin reduced and embedded.

*Fins*. Dorsal fin with 3–4 unbranched rays and 7 branched rays, origin slightly posterior to pelvic fin origin. Tip of adpressed dorsal fin reaches within 2–3 scales to vertical through posterior base of anal fin, distal margin straight. Pectoral fin with 13–15 rays, fan shaped, larger in males than females, tip of adpressed pectoral fin reaches and surpasses base of pelvic fin in males, reaches 2 scales to base of pelvic fin in females. Pelvic fin with 8–9 rays, origin slightly in front of dorsal fin origin, outer margin slightly convex, its tip reaching anterior origin of anal fin when depressed in males and reaches 2–3 scales to anal fin origin in females. Anal fin with 3 simple rays and 5 branched rays, distal margin almost straight to slightly convex or straight, origin closer to anterior base of pelvic fin than base of caudal fin. Caudal fin is forked, with 10+9 principal rays.

*Osteology*. Total vertebrae including Weberian apparatus 35–38 (mode 37), predorsal vertebrae including Weberian apparatus 11–13 (mode 12), precaudal vertebrae including Weberian apparatus 18–19 (mode 19), caudal vertebrae including Weberian apparatus 16–18 (mode 18).

*Colouration (live and fresh specimens)*. Refer to Figure 5 for general live colouration. Dorsum and sides dark brown, belly and underparts off-white or silvery, operculum metallic gold, base of fins bright red. Vague lateral stripe terminating in a triangular blotch at the base of the caudal fin.

*Colouration (preserved)*. Dorsal surface of alcohol preserved specimens dark grey or black, sides and belly lighter. Distinct black lateral stripe from posterior margin of operculum terminating into a black triangular blotch of pigment at the base of the caudal peduncle. Red pigmentation at the base of fins disappears in preserved specimens (Figure 6b).

##### Distribution.

*Pseudobarbus senticeps* (referred to as the ‘Krom lineage’ by Swartz et al. 2007, 2009) is endemic to the Krom River system which discharges into St Francis Bay (Figure 7). The species has a restricted known distribution range, surviving in a few relatively unimpacted and uninvaded tributaries of the Krom River system (Figure 7).

##### Habitat.

*Pseudobarbus senticeps* inhabits perennial mountain streams with clear to peat stained water, cobble and pebble substrates.

##### Conservation status.

*Pseudobarbus senticeps* (referred to as *Pseudobarbus* sp. “afer Krom” by Tweddle et al. 2009) was listed as Critically Endangered during the IUCN assessment of the status and distribution of freshwater fishes in southern Africa (Tweddle et al. 2009). Invasion by alien fish (particularly *Micropterus* spp.) was identified as the major threat to this species. Further studies are required to more accurately assess the distribution, ecology and biology of this species.

#### Pseudobarbus swartzi sp. n.

Taxon classificationAnimaliaTeleosteiCyprinidae

http://zoobank.org/CCA9F17C-F36C-4B48-BAD9-C5AEA3B6D161

Figures 5c , 6d

##### Proposed common name.

Gamtoos redfin.

##### Holotype.

SAIAB 203792 (Field no: AC15AL39), male, 80.9 mm SL, Tributary of the Wabooms, Gamtoos River system, -33.8639772 S, 23.8263333E, collected by A Chakona, B Motshegoa, N Mazungula, W Kadye and R Smith, 21 January 2015.

##### Paratypes.

SAIAB 203793 (Field no: AC15AL39), 9 unsexed, 35.4–76.0 mm SL, same locality information and collectors as holotype; MRAC 2016-032-P-0001-0004 (Field no: AC16AL02), 4 unsexed, 50.2–61.4 mm SL, main tributary of the Louterwater River, -33.8333611S, 23.6373056E, Gamtoos River system, collected by A Chakona, S Reddy and R Smith, 18 January 2016.

##### Etymology.

The species is named after Dr Ernst R. Swartz for his contribution to the biogeography and systematics of *Pseudobarbus* and the role that he played in mentoring students working on the systematics and biogeography of southern African freshwater fishes.

##### Diagnosis.

Possession of a single pair of oral barbels separates *Pseudobarbus swartzi* sp. n. from *Pseudobarbus burchelli, Pseudobarbus burgi, Pseudobarbus skeltoni and Pseudobarbus verloreni* all with two pairs. It differs from *Pseudobarbus quathlambae* by having larger scales and fewer scale rows along the lateral line (*Pseudobarbus swartzi*: 35–37, mode 35–36 lateral line scales; *Pseudobarbus quathlambae*: > 65 scales along lateral line). *Pseudobarbus swartzi* and *Pseudobarbus senticeps* show some overlap in barbel length (Figure 4), but are clearly separated by scale size (Figure 4). *Pseudobarbus swartzi* has a higher number of scale rows along the lateral line (34–37, mode 36) *vs* (25–33, mode 29), and around the caudal peduncle (13–17, mode 16) *vs* (10–12, mode 11) in *Pseudobarbus senticeps*. *Pseudobarbus swartzi* further differs from *Pseudobarbus senticeps* by lacking a conspicuous lateral stripe which terminates in a triangular blotch at the base of the caudal fin. While there is some overlap in scale counts between *Pseudobarbus swartzi* and *Pseudobarbus afer*, it is uncommon (see above). *Pseudobarbus swartzi* has a higher number of lateral scale series (34–37, mode 36) than *Pseudobarbus afer* s.s which has fewer lateral scale series (29–35, mode 32). The two species further differ in scale pigmentation pattern (see Figure 6a, 6). *Pseudobarbus afer* has a band of pigment along the centre of the scales, leaving a clear band along the distal edges of the scale, and producing a distinct mesh or net-like pattern which is more conspicuous on the latero-ventral scales. This pattern is not evident in *Pseudobarbus swartzi*. *Pseudobarbus swartzi* sp. n. differs from *Pseudobarbus asper* by possession of fewer larger scales (lateral line scale series 34–37, mode 36 *vs* 35–45, mode 37–40; caudal peduncle scale rows 13–17, mode 16 *vs* 16–22, mode 18–20). Lack of prominent black spots and blotches on the body distinguishes *Pseudobarbus swartzi* from *Pseudobarbus phlegethon*.

##### Description.

General appearance and colouration is shown in Figs 5c, 6d. Morphometric and meristic data summarised in Table 4 are based on 64 specimens (43.6 - 81.1 mm SL) collected from 11 localities across the Gamtoos River system. A fusiform minnow with body slightly compressed laterally. Predorsal profile convex, post dorsal profile straight and descending from origin of dorsal fin to caudal fin insertion. Ventral profile more or less straight from tip of snout to pelvic fin origin. Head relatively short, length almost equal to body depth, its dorsal profile distinctly convex, particularly from its tip to interorbital area. Mouth subterminal, its corner not reaching vertical through anterior margin of eye. Barbels attached from behind the rictus of the mouth, barbel length variable, with some individuals having long barbels reaching or surpassing vertical through posterior edge of the eye, while other individuals have much shorter barbels (see Figure 4c). The eye is located closer to the tip of the snout than to posterior edge of the operculum, eye diameter shorter than snout length. Snout blunt and moderately rounded.

*Tuberculation*. Mature breeding males develop large conical tubercles on the snout and along the dorsal edge of the nares and eyes. Bilateral clusters on snout include 2–4 tubercles in mature ripe males. Smaller, scattered tubercles develop on the head dorsum. Bands of fine tubercles along dorsal surface of pectoral fin rays.

*Scales*. Scale rows along lateral line 34–37 (mode 36) ending at hypural, with 1–2 more scales to base of caudal fin; 6–7 (mode 6) scale rows between lateral line and dorsal fin origin; 4–5 (mode 5) rows between lateral line and pelvic fin origin, 5 rows between lateral line and anal fin origin, 16–20 (mode 17–18) pre-dorsal scale rows, 13–17 (mode 16) scale rows around caudal peduncle. Triangular naked patch between the gill covers and anterior base of pectoral fins, scales between pectoral fin origin and pelvic fin origin reduced and embedded. Axillary scales of pelvic fin not prominent or elongate. Scales between posterior edge of head and dorsal fin origin embedded and smaller than flank scales.

*Fins*. Dorsal fin is inserted about mid-body (excluding caudal fin), origin slightly behind vertical through origin of pelvic fin, with 3–4 unbranched rays and 7 branched rays, distal margin straight to slightly, posterior tip of depressed dorsal fin does not reach vertical through posterior base of anal fin. Pectoral fins fan-shaped, larger in males than females, with 14–16 rays, tip of depressed pectoral fin almost overlapping with pelvic fin in large males, reaches 2 scales to base of pelvic fin in females. Pelvic fin with 8 rays, origin slightly in front of dorsal fin origin, tip of depressed pelvic fin not reaching anterior origin of anal fin, except in mature males. Anal fin with 3 simple rays and 5 branched rays, distal margin straight to slightly convex, origin closer to anterior base of pelvic fin than caudal fin base. In mature males, tip of depressed pelvic fin often surpass point of anal fin origin while they only reach up to the anus in females. Caudal fin is forked, with 10+9 principal rays.

*Colouration (live and fresh specimens)*. Refer to Figure 5c for general live colouration. Body golden-tan laterally, becoming darker dorsally, and lighter to white ventrally. Base of fins bright red, operculum metallic gold.

*Colouration (preserved)*. Alcohol preserved specimens are dark greyish above the lateral line, light grey or whitish below the lateral line and ventrally, breast of freshly preserved specimens silvery (Figure 6). Mid lateral stripe present but comparatively obscure, black blotch at the base of caudal fin inconspicuous.

*Osteology*. Total vertebrae including Weberian apparatus 37–38 (mode 37), predorsal vertebrae including Weberian apparatus 12–13 (mode 13), precaudal vertebrae including Weberian apparatus 19–20 (mode 20), caudal vertebrae including Weberian apparatus 17–18 (mode 18).

##### Distribution.

*Pseudobarbus swartzi* sp. n. occurs in the Kougaberg, Baviaanskloofberg and Elandsberg tributaries of the Kouga and Groot sub-catchments of the Gamtoos River system, and the Kabeljous and Swart River systems which discharge into St Francis Bay (Figure 7). Remnant populations in the Kouga and Groot sub-catchments are highly fragmented due to invasion of the main stem sections of the rivers by alien predators in particular the African sharptooth catfish (*Clarias gariepinus*) and the North American black bass species (*Micropterus* spp.). The status and distribution of *Pseudobarbus swartzi* populations in the Kabeljous and Swart river systems need to be assessed through fine scale geographic surveys. There is also need for investigations to determine the taxonomic status of redfins in the Seekoei and Maitland, two river systems which also discharge into St Francis Bay (Figure 7).

##### Habitat.

*Pseudobarbus swartzi* inhabits perennial mountain streams with clear or peat stained water as well as cobble, pebbles and boulders.

##### Conservation status.

Remnant populations of the species are under severe threat from multiple human impacts including habitat degradation, complete water abstraction and potential invasion by alien fish predators and competitors that are now dominant in mainstem sections of the rivers (Ellender et al. 2011, 2015). *Pseudobarbus swartzi* sp. n. (referred to as *Pseudobarbus* sp. “afer Gamtoos” by Tweddle et al. 2009) was listed as Endangered following the IUCN assessment of the status and distribution of freshwater fishes in southern Africa (Tweddle et al. 2009). Invasive alien fishes were identified as the single most important threat to this species.

### Other material examined

*Pseudobarbus afer*: SAIAB 34422, 5 males (44.9–65.5 mm SL), 5 females (59.2–74.5 mm SL), Blindekloof River, Groendal Wilderness, Swartkops River system, collected by D Boulle and PH Skelton, 11 November 1988; SAIAB 34428, 5 unsexed (60.1–75.1 mm SL), Blindekloof River, Groendal Wilderness, Swartkops River system, collected by D Boulle, 8 June 1989; SAIAB 121688 (formerly AMG 2524), 24 unsexed (46.0–81.0 mm SL), Elands River, Swartkops River system, -33.7667S, 25.1278E, collected by PH Skelton and A Bok, 5 September 1974; SAIAB 119909 (formerly AMG745), 5 unsexed (46.0–61.0 mm SL), Elands River, Swartkops River system, -33.71667S, 25.1000E, collected by RA Jubb, 15 February 1964; SAIAB 119773 (formerly AMG 609), 30 unsexed (48.5–66.5 mm SL), Wit River, Sundays River system, -33.3333333S, 25.6833333E, collected by R A Jubb, 8 April 1959. SAIAB 119940 (formerly AMP 776), 5 unsexed (43.0–82.0 mm SL), Kragga Kamma, Baakens River system, -33.9500000S, 25.5000000E, collected by D Bicknell, 15 January 1964.

*Pseudobarbus swartzi*: AC16AL01 (SAIAB 203772), 10 specimens, unsexed, 25.5–57.9 mm SL, western Tributary of the Louterwater River, -33.8257500S, 23.6310000E, Gamtoos River system, collected by A Chakona, S Reddy and R Smith, 18 January 2016; AC16AL02 (SAIAB 203779), 6 specimens, unsexed, 32–64.8 mm SL, main Tributary of the Louterwater River, -33.8333611S, 23.6373056E, Gamtoos River system, same collectors and date as AC16AL01 (SAIAB 203772); AC16AL04 (SAIAB 203787), 34 specimens, unsexed, 18.2–86.7 mm SL, upper Dwars River, -33.6534444S, 23.7539722E, Gamtoos River system, same collectors and date as AC16AL01; AC16AL05 (SAIAB 203786), 17 specimens, unsexed, 34.8–64.9 mm SL, Klein River at Kouga Wilderness, -33.7112222S, 23.8440833E, Gamtoos River system, same collectors as AC16AL01 (SAIAB 203772), 19 January 2016; AC16AL06 (SAIAB 203789), 8 specimens, unsexed, 47.8–70.2 mm SL, Braam River, -33.7135278S, 23.8465833E, Gamtoos River system, same collectors as AC16AL01 (SAIAB 203772), 19 January 2016; AC16AL07 (SAIAB 203788), 13 specimens, unsexed, 17.9–63.3 mm SL, Diep River, -33.7541944S, 24.0812500E, Gamtoos River system, A Chakona and R Smith, 20 January 2016; AC16AL08 (SAIAB 203781), 45 specimens, unsexed, 14.7–53.8 mm SL, Upper Kansenkei River, -33.7296667S, 24.5545833E, Gamtoos River system, same date and collectors as AC16AL07 (SAIAB 203788); AC16BL01 (SAIAB 203774), 10 specimens unsexed, 25.5–57.9 mm SL, Wit River, -33.6538333S, 24.51605556E, Gamtoos River system, A Chakona and B Motshegoa, 7 March 2016; AC16BL02 (SAIAB 203780), 5 specimens unsexed, 24.6–58.8 mm SL, Lourie River, -33.8506944S, 25.0388194E, Gamtoos River system, A Chakona and B Motshegoa, 7 March 2016; SAIAB 120538 (formerly AMG1374), 70 unsexed, Kouga Dam, Gamtoos River system, -33.6666667S, 24.5166667E, collected by F Farquharson, 6 July 1967; SAIAB 120539, 70 unsexed, same locality and collector as SAIAB 120538.

## Discussion

### Comparative remarks

The three species recognised in the present study exhibit subtle morphological differences and show marginal overlap in some meristic (i.e. scale) counts. This explains why these species were previously considered to represent one widespread but variable species (Skelton 1988). Use of molecular data and careful examination of morphometric and meristic data in the present study helped to reveal consistent genetic, scale count, oral barbel length and colour pattern differences among Swartz et al.’s (2007) Mandela, St Francis and Krom lineages, supporting their recognition as distinct species. We redescribed *Pseudobarbus afer* s.s (Mandela lineage), resurrected *Pseudobarbus senticeps* (Krom lineage) and described a new species *Pseudobarbus swartzi* (St. Francis lineage). The three species are endemic to the streams of the eastern Cape Fold Ecoregion (CFE) at the south-eastern tip of Africa where they are allopatrically distributed.

These morphologically very similar species can be distinguished based on a combination of lateral line scale counts, circumpeduncular scale counts, body colour pattern and length of oral barbels. *Pseudobarbus senticeps* differs from both *Pseudobarbus afer* and *Pseudobarbus swartzi* by having fewer (i.e. larger) scales along the lateral line (mode 29), fewer scales around the caudal peduncle (mode 11) and a distinct lateral stripe that terminates in a triangular blotch at the base of the caudal fin. This colour pattern becomes more pronounced in preserved specimens. *Pseudobarbus senticeps* further differs from *Pseudobarbus afer* by having distinctly long barbels that reach or surpass vertical through the posterior edge of the eye. Barbels and other external features such as fins are however susceptible to degradation particularly in habitats affected by waste water discharge. Caution should therefore be exercised, and it is recommended that identification of the species should not be based on barbel length alone, but should be used in combination with scale counts and colour pattern. *Pseudobarbus swartzi* has smaller scales, i.e. more scales along the lateral line (mode 36) and around the caudal peduncle (mode 16) compared to *Pseudobarbus afer*. (mode 32 and 12, respectively). *Pseudobarbus swartzi* and *Pseudobarbus afer* further differ in scale pigmentation pattern. In *Pseudobarbus afer*, the melanophores form a semi-circular band around the centre of the scale, while the basal segment of the exposed area and the distal margin are not pigmented. This produces a distinct mesh or net-like pattern which is more pronounced on the latero-ventral scales (see Figure 6). This pigmentation pattern is not evident in *Pseudobarbus swartzi*.

The close morphological similarity observed among the three allopatric and genetically divergent species reported in the present study has also been reported for other riverine fishes, including the African butterfly fish, *Pantodon buchholzi*, from the Niger and Congo river systems (Lavoué et al. 2010) and the dwarf loach, *Cobitis brevifasciata* (previously *Kichulchoia brevifasciata*), from the Goheung Peninsula in South Korea (Kim et al. 2013). A combination of mechanisms, including stabilising selection (see Bickford et al. 2007), genetic variation and developmental constraints as well as ecological niche conservatism (Erwin 2007) have been proposed as possible explanations for the absence of significant accrual of species-wide morphological change over long evolutionary time scales (morphological stasis) observed in both extant taxa (e.g. Moen et al. 2013) and the fossil record (see Eldredge et al. 2005). *Pseudobarbus afer*, *Pseudobarbus swartzi* and *Pseudobarbus senticeps* inhabit mountain streams which are very similar ecologically. These streams which are characterised by clear acidic waters and rocky substrates have been classified as harsh environments by Dallas and Rivers-Moore (2014) as they experience extreme fluctuations in water temperature and flows between winter and summer months. According to Bickford et al. (2007), organisms that inhabit extreme environments may be more prone to stabilising selection which reduces or eliminates species-wide morphological change, because “there are a limited number of ways in which an organism can adapt to harsh conditions”. Thus, ecological niche conservatism (due to occurrence in similar habitats) and stabilising selection (occurrence in extreme environmental conditions) are both possible mechanisms that could explain the lack of clear morphological differentiation among *Pseudobarbus afer*, *Pseudobarbus senticeps* and *Pseudobarbus swartzi* sp. n., but these hypotheses require further testing with other co-distributed stream fishes. The ongoing discovery of extreme levels of cryptic diversity within other genera of stream fishes endemic to the CFE such as *Galaxias* (Waters and Cambray 1997; Wishart et al. 2006; Chakona et al. 2013a) and *Sandelia* (Chakona et al. 2013a) suggests that morphological conservatism may be prevalent among stream fishes of the CFE. This region thus presents a particularly promising opportunity to undertake comparative studies to investigate the intrinsic and extrinsic mechanisms that are involved in maintaining morphological stasis in allopatric and genetically divergent stream fishes over varying evolutionary time scales.

### Overall biogeographic patterns

**Figure 8.**
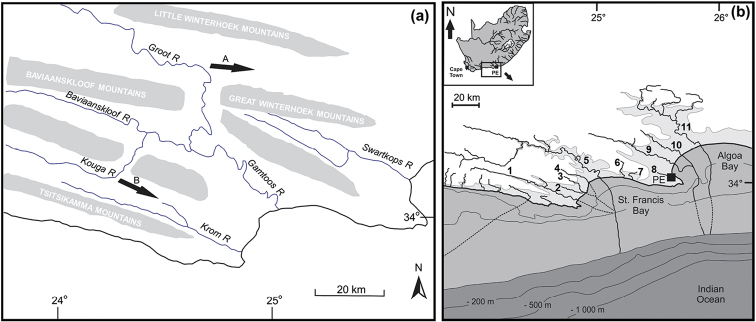
**a** An illustration of part of the Cape Fold Belt showing the drainage of the Gamtoos River system, sites of drainage capture of adjacent river systems and historical direction of flow of captured rivers (modified from Skelton, 1980) **b** Part of the eastern Cape Fold Ecoregion showing reconstructed Palaeoriver systems during the Last Glacial Maximum (modified from Swartz et al., 2007). The numerals represent present day river systems in the study area for the present study: 1, Krom; 2, Seekoei; 3, Swart; 4, Kabeljous; 5, Gamtoos; 6, Van Stadens; 7, Maitland; 8, Baakens; 9, Swartkops; 10, Coega; 11, Sundays).

The CFE experienced a complex history that left a perceptible imprint in the distribution and diversity of stream fishes in the region (see Skelton 1980; Swartz et al. 2007, 2009, 2014; Chakona et al. 2013a,b; Chakona et al. 2015). River captures and marine transgressions are likely to be the primary mechanisms that drove diversification and shaped the distribution patterns of the three redfins (*Pseudobarbus afer*, *Pseudobarbus senticeps* and *Pseudobarbus swartzi*) that are endemic to the eastern CFE. The geomorphology of this region (see Figure 8a) indicates that the drainages of the eastern CFE were influenced by a series of complex river captures (Haughton et al. 1937). According to Haughton et al. (1937), the Groot River historically flowed down the strike valley between the Great and Little Winterhoek mountains (arrow A in Figure 8a), probably forming a section of the headwaters of the Sundays River system. The Kouga River is thought to have formerly flowed through the strike valley between the Tsitsikamma and Baviaanskloof mountains (arrow B in Figure 8), probably forming the headwaters of the Krom River system. Both the Kouga and Groot Rivers abruptly cut across major mountain ranges to join the Baviaanskloof River (Figure 8a), suggesting that they were captured by the Gamtoos River system. These events could have had an influence on the diversification and present day distribution of redfins in the eastern CFE, but the specific role of river captures cannot be evaluated at this stage as no dating estimates exist for these events. More surveys are required to determine the extent of distribution of *Pseudobarbus swartzi* in the Groot catchment.

Sea-level changes offer another alternative explanation for the observed genetic and distribution patterns of redfins in the eastern CFE. Palaeoriver reconstructions for this region show that the Pliocene marine transgression resulted in fragmentation of the major river systems (the Krom, Gamtoos, Swartkops and Sundays), while smaller coastal systems (e.g. the Swart, Seekoei, Kabeljous, Van Stadens, Maitland, Baakens and Coega) were drowned and were presumably unavailable to freshwater taxa during this period (see Swartz et al. 2007; Figure 8b). Assuming the cytochrome *b* mutation rate of 2% per Myr (see Thorpe et al. 2005), age estimates for the splitting between *Pseudobarbus afer*, *Pseudobarbus senticeps* and *Pseudobarbus swartzi* coincide with the Pliocene sea-level transgression, suggesting that isolation of *Pseudobarbus* populations in upland refugia in the Krom, Gamtoos and the Swartkops or Sundays could have played a role in promoting divergence and speciation of these redfins, as proposed for other stream fishes in the south-western CFE (Chakona et al. 2013a). Occurrence of *Pseudobarbus swartzi* in the currently isolated Gamtoos, Kabeljous and Swart River systems is consistent with expectations of post-speciation range expansion that is likely to have been facilitated by confluence of these rivers during the Last Glacial Maximum (LGM) low sea-levels (Swartz et al. 2007, 2009; Figure 8b). However, the lack of sharing of species between the Krom and the Gamtoos-Swart-Kabeljous is not consistent with the Palaeoriver hypothesis, as map reconstructions suggest that these four river systems would have joined before reaching the -130 m LGM sea-level (Swartz et al. 2007, 2008; Figure 8b). Presence of instream physical barriers such as waterfalls or extreme ecological barriers could have prevented post-speciation range expansion between these systems, as proposed for the Breede and Heuningnes lineages of *Pseudobarbus burchelli* from the south-western CFE (Swartz et al. 2014). Palaeoriver reconstructions suggest that the Swartkops-Coega-Baakens would have formed a common confluence before reaching the -130 m LGM sea-level, but it is uncertain whether this Palaeoriver system would have coalesced with the Sundays River system (see Swartz et al. 2007; Figure 8b) due to the intervening Riy Bank (Bremner and Day 1991). If the Sundays remained isolated from the Swartkops-Coega-Baakens Palaeoriver system, a recent river capture event between the Swartkops and Sundays could provide an alternative explanation for the occurrence of *Pseudobarbus afer* in both systems. Presence of instream physical barriers or extreme ecological gradients could explain the absence of *Pseudobarbus afer* from the Coega River system which is inferred to have coalesced with the adjacent Swartkops and Baakens River systems during the LGM low sea-levels.

### Conservation concerns

As with many other endemic stream fishes in the CFE, there is need for immediate intervention measures to ensure future survival of *Pseudobarbus afer*, *Pseudobarbus swartzi* and *Pseudobarbus senticeps*. As with elsewhere in the CFE (see Clark et al. 2009), *Pseudobarbus afer*, *Pseudobarbus swartzi* and *Pseudobarbus senticeps* have suffered severe range reductions mainly due to introduction of non-native predators, habitat alteration, complete water abstraction and building of weirs (Tweddle et al. 2009; Ellender et al. 2011, 2015; Ellender and Weyl 2014). Remnant populations of *Pseudobarbus afer*, *Pseudobarbus swartzi* and *Pseudobarbus senticeps* are highly fragmented and now only persist in upper mountain tributaries that have not been heavily degraded or invaded by alien species. There are limited or no opportunities for genetic exchange between isolated populations which can reduce the reproductive fitness and long-term evolutionary flexibility and adaptive responses of these species in the face of projected environmental changes in the region (Dallas and Rivers-Moore 2014). *Pseudobarbus afer*, *Pseudobarbus swartzi* and *Pseudobarbus senticeps* were listed under highly threatened categories of the IUCN during the previous assessment of the status of freshwater systems in southern Africa (see Tweddle et al. 2009). *Pseudobarbus afer* (identified as *Pseudobarbus afer* by Tweddle et al. 2009) and *Pseudobarbus swartzi* (identified as *Pseudobarbus* sp. “afer Gamtoos” by Tweddle et al. 2009) were both listed as Endangered, while *Pseudobarbus senticeps* (identified as *Pseudobarbus* sp. “afer Krom” by Tweddle et al. 2009) was listed as Critically Endangered. Ongoing decline is likely, and other populations (for example in the Baakens, Swart and Kabeljous river systems) are feared to have been extirpated or may be represented by only a few individuals as redfins were not collected from these systems during recent surveys. *Pseudobarbus senticeps* needs to be prioritised as it is represented by very few remaining populations with very small known population sizes. Eradication of non-native fishes and control of effluent discharge are the immediate conservation actions required to secure remaining populations of *Pseudobarbus senticeps*. Additional fine scale field surveys are required to determine the status of redfin populations in the Baakens, Van Stadens, Swart and Kabeljous river systems. Future studies should aim to examine the ecology of the three species identified in the present study and model their potential responses to present and future environmental changes that are projected to impact biotic communities of the Cape Fold Ecoregion.

## Supplementary Material

XML Treatment for Pseudobarbus afer

XML Treatment for Pseudobarbus senticeps

XML Treatment for Pseudobarbus swartzi
